# Pregnancy-related disorders: molecular mechanisms and management

**DOI:** 10.1186/s43556-026-00566-6

**Published:** 2026-10-12

**Authors:** Chehao Lee, Chan Zhu, Yang Li, Jiao Yang, Xuan Zhang, Wen Jiang, Deying Mou, Huafeng Li, Qian Hu

**Affiliations:** 1 Department of Traditional Chinese Medicine, West China Second University Hospital, Sichuan University, Chengdu, 610041 China; 2 Laboratory of Molecular Translational Medicine, Centre for Translational Medicine, West China Second Hospital, Sichuan University, Chengdu, Sichuan 610041 China; 3 Department of Gynecology and Obstetrics, West China Second Hospital, Sichuan University, Chengdu, Sichuan 610041 China; 4 Key Laboratory of Birth Defects and Related Diseases of Women and Children, Ministry of Education, Clinical Research Center for Birth Defects of Sichuan Province, West China Second Hospital, Sichuan University, Chengdu, Sichuan 610041 China; 5 Department of Respiratory, Chengdu Qingyang Hospital of Traditional Chinese Medicine, Chengdu, 610072 China; 6 Department of Anesthesiology, West China Women’s and Children’s Hospital, Sichuan University, Chengdu, 610041 China

**Keywords:** Pregnancy-related disorders, Placental-maternal adaptation failure continuum, Two-hit framework, Mechanism-informed therapeutics, Pregnancy memory

## Abstract

Pregnancy-related disorders (PRDs), including preeclampsia (PE), gestational diabetes mellitus (GDM), hyperemesis gravidarum (HG), and intrahepatic cholestasis of pregnancy (ICP), remain major causes of maternal and perinatal morbidity. This review synthesizes their molecular, endocrine, immune, and clinical features. We propose the Placental-Maternal Adaptation Failure Continuum (PMAFC), in which gestational/fetoplacental stress load interacts with maternal adaptive reserve. This load includes placenta-derived signals, the physiological demands of gestation and fetal growth, and secondary maternal or fetal mediators. Placental drive denotes the placenta-derived component, whereas maternal adaptive reserve reflects the capacity of vascular, metabolic, hepatic, neuroendocrine, and immune systems to compensate for pregnancy-related challenges. We distinguish experimentally derived mechanistic hypotheses from findings supported by human studies and consider persistent post-pregnancy changes, termed pregnancy memory, as a potential link to later maternal cardiometabolic risk. We also separate established care from investigational biomarkers and interventions. The soluble fms-like tyrosine kinase-1/placental growth factor (sFlt-1/PlGF) ratio can support clinical assessment in selected PE pathways. Cell-free RNA, chromosome 19 microRNA cluster biomarkers, and polygenic risk scores remain investigational. Apheresis has limited human proof-of-concept evidence, whereas sFlt-1 silencing, growth differentiation factor 15 receptor blockade, and strategies to restore hepatobiliary transporter function remain under clinical or preclinical evaluation. PMAFC is an evidence-organizing framework, not a validated predictive or therapeutic algorithm.

## Introduction

Pregnancy requires coordinated immune, metabolic, cardiovascular, and hepatobiliary adaptation to support placental and fetal development [1, 2]. Failure of these responses can contribute to pregnancy-related disorders (PRDs), including preeclampsia (PE), gestational diabetes mellitus (GDM), hyperemesis gravidarum (HG), and intrahepatic cholestasis of pregnancy (ICP) [3–6]. Placenta-mediated conditions such as PE are often discussed within the great obstetrical syndromes, which link placental pathology to maternal systemic responses and adverse outcomes [3, 7]. Although disease burden varies across populations and diagnostic criteria, PE and GDM are common, whereas HG and ICP are less prevalent but can cause severe maternal or fetal morbidity [3–6]. Despite this heterogeneity, current management remains disease-specific and includes established screening, symptom control, maternal and fetal surveillance, and delivery when indicated. These approaches are essential but do not fully address biological heterogeneity or every upstream mechanism. Epidemiological and mechanistic overlap between GDM and PE suggests partly shared vascular, metabolic, and inflammatory pathways [8, 9]. These associations do not establish a common causal sequence. A placenta-only interpretation is also insufficient because placental pathology interacts with maternal vascular susceptibility [10–13]. Genetic and epigenetic factors may further modify PE and GDM phenotypes [14–16].

The Placental-Maternal Adaptation Failure Continuum (PMAFC) addresses this interpretive gap by comparing patterns of maternal-placental maladaptation while preserving disease-specific diagnostic boundaries. This review uses PE, GDM, HG, and ICP as representative, rather than exhaustive, examples. The framework interprets each phenotype through the interaction between a disorder-relevant gestational/fetoplacental stress load (Hit 1) and maternal adaptive reserve (Hit 2) [3, 12, 17]. Hit 1 includes placenta-derived signals, physiological gestational and fetal demands, and secondary maternal or fetal mediators. Placental drive denotes only its placenta-derived subset. Hit 2 denotes vulnerability arising from limited capacity within vascular, metabolic, hepatic, neuroendocrine, or immune systems to buffer these challenges. The two hits interact dynamically rather than sequentially. A challenge may remain compensated when the relevant reserve is sufficient and become maladaptive when reserve is limited.

The composition of Hit 1 and the relevant domain of Hit 2 differ among disorders. In PE, Hit 1 is predominantly placenta-derived and includes anti-angiogenic and inflammatory signals, whereas Hit 2 reflects vulnerability arising from limited vascular and endothelial adaptive reserve [17–19]**.** In GDM, Hit 1 comprises placental endocrine and inflammatory signals that interact with broader gestational metabolic demands, whereas Hit 2 reflects limited metabolic and β-cell compensatory reserve [20, 21]. In HG, fetoplacental growth differentiation factor 15 (GDF15) exposure is a major component of Hit 1, whereas Hit 2 includes variation in maternal GDF15 sensitivity and the capacity to maintain nutritional homeostasis [22]. In ICP, Hit 1 comprises the effects of gestational hormones and their metabolites on maternal hepatobiliary transporter expression and function, whereas Hit 2 reflects limited hepatobiliary transport reserve [23–25]. Their bioactive properties may also produce context-dependent systemic effects, although this possibility remains hypothesis-generating [26, 27].

PMAFC also incorporates the hypothesis of pregnancy memory. This term describes persistent molecular, cellular, or structural changes after a complicated pregnancy [28, 29]. Candidate processes include epigenetic alteration, maladaptive vascular repair, persistent remodeling, and trained-immunity-like inflammatory reprogramming [28, 30–32]. These processes may contribute to later cardiovascular and metabolic risk after PE or GDM [33–35]. However, pre-existing susceptibility and shared risk factors remain important alternative or complementary explanations.

This review first outlines the cellular and molecular adaptations that support healthy pregnancy. It then examines the mechanisms of PE, GDM, HG, and ICP. We next introduce PMAFC as a framework for organizing gestational/fetoplacental stress load and maternal adaptive reserve. Later sections stratify biomarkers and interventions by evidence level, separating established care from emerging approaches. Throughout, we distinguish established evidence from mechanistic inference and hypothesis-generating cross-disorder associations. We conclude with postpartum surveillance and long-term maternal health.

## The cellular and molecular physiology of pregnancy: a systemic stress test

Understanding PRDs first requires defining the physiological range of pregnancy adaptation. Maternal organ systems undergo coordinated changes that expand vascular, metabolic, hepatic, neuroendocrine, and immune capacity [1]. These changes involve increased organ function and recalibration of homeostatic set points [36]. Pregnancy can therefore act as a biological stress test that reveals latent limitations in maternal organ reserve [12]. This section summarizes the cellular and molecular architecture of these adaptations (Fig. 1).

**Fig. 1:**
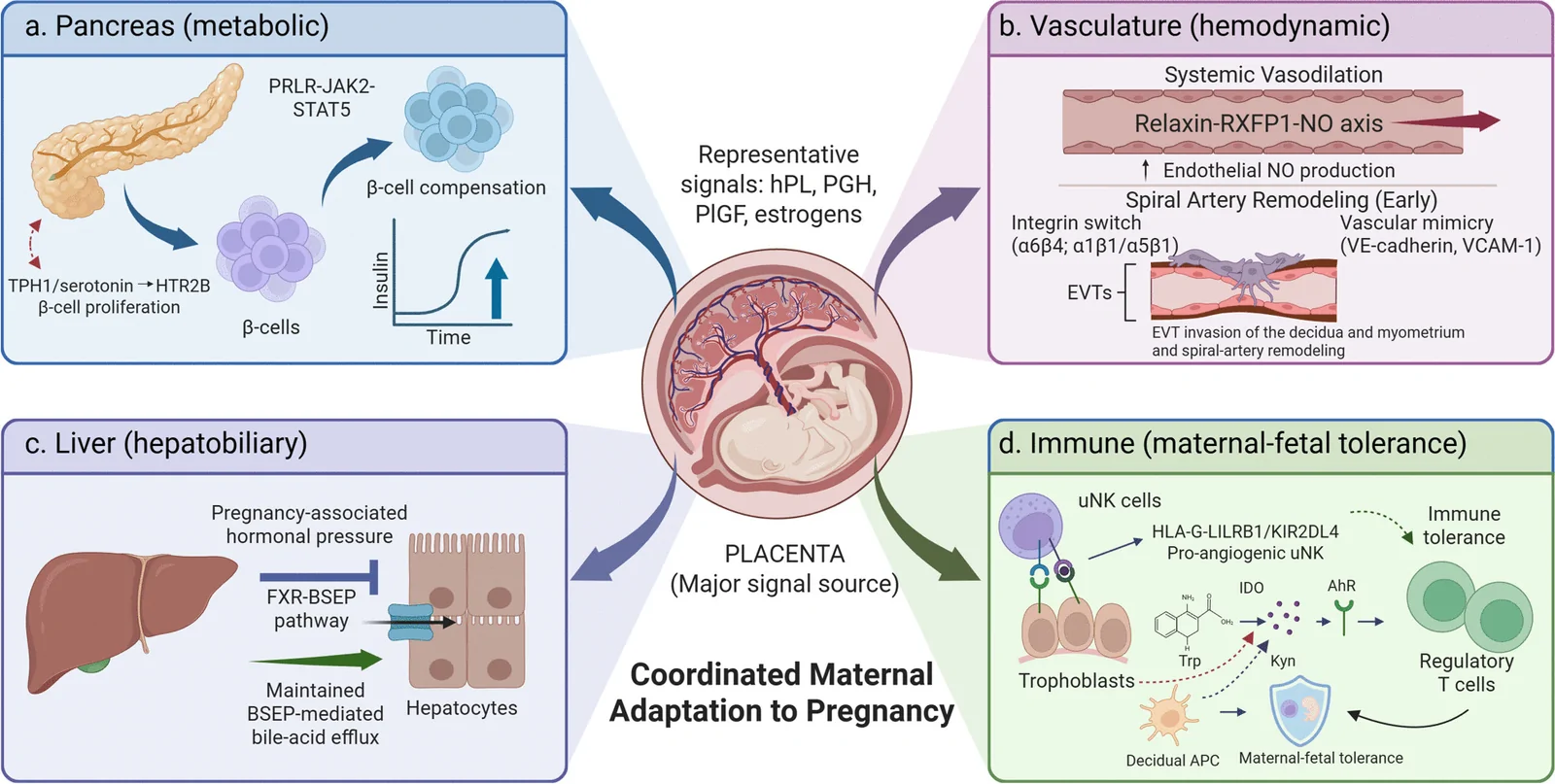
Multi-organ adaptation during healthy pregnancy. Placenta-derived and pregnancy-associated signals recalibrate maternal physiological set points and coordinate pancreatic, vascular, hepatobiliary and immune adaptation. **a** Lactogenic signaling through PRLR-JAK2-STAT5 supports pancreatic β-cell compensation during pregnancy. The TPH1-serotonin-HTR2B proliferative pathway is supported predominantly by rodent studies. **b** Relaxin-RXFP1-NO signaling promotes systemic vasodilation, whereas EVT invasion and spiral artery remodeling support the establishment of a low-resistance uteroplacental circulation. **c** Hepatobiliary adaptation maintains BSEP-mediated bile acid export despite pregnancy-associated hormonal pressure on FXR-regulated canalicular transport. **d** Trophoblast HLA-G interactions with LILRB1 and KIR2DL4 shape uNK-cell functions, including pro-angiogenic responses. IDO, expressed by trophoblasts and decidual APCs, catalyzes tryptophan-to-kynurenine metabolism, and downstream AhR signaling supports Treg differentiation. Together, these complementary decidual immune circuits contribute to context-specific maternal–fetal tolerance. Abbreviations: AhR, aryl hydrocarbon receptor; APC, antigen-presenting cell; BSEP, bile salt export pump; EVT, extravillous trophoblast; FXR, farnesoid X receptor; HLA-G, human leukocyte antigen G; hPL, human placental lactogen; HTR2B, 5-hydroxytryptamine receptor 2B; IDO, indoleamine 2,3-dioxygenase; JAK2, Janus kinase 2; KIR2DL4, killer cell immunoglobulin-like receptor 2DL4; Kyn, kynurenine; LILRB1, leukocyte immunoglobulin-like receptor B1; NO, nitric oxide; PGH, placental growth hormone; PlGF, placental growth factor; PRLR, prolactin receptor; RXFP1, relaxin family peptide receptor 1; STAT5, signal transducer and activator of transcription 5; TPH1, tryptophan hydroxylase 1; Treg, regulatory T cell; Trp, tryptophan; uNK, uterine natural killer cell. The figures were created using BioRender.com

### Metabolic reprogramming: physiological insulin resistance

Pregnancy has a biphasic metabolic profile. An early anabolic phase, approximately 0–20 weeks, promotes maternal lipid storage. A later catabolic phase, approximately 20–40 weeks, includes physiological insulin resistance that facilitates fetal nutrient supply [37].

#### Molecular basis of maternal insulin resistance

By the third trimester, maternal insulin sensitivity decreases by approximately 50%–70% [21]. This physiological change supports fetal nutrient transfer but differs from the persistent insulin resistance of type 2 diabetes mellitus. Human placental lactogen (hPL) and placental growth hormone (PGH) increase with placental mass and contribute to this adaptation [38]. Post-receptor insulin signaling is reduced in skeletal muscle and adipose tissue, including lower IRS-PI3K-Akt pathway activity [21]. Free fatty acids and tumor necrosis factor-α (TNF-α) can activate serine kinases such as JNK, IKKβ, and PKCδ [39]. These kinases inhibit insulin receptor substrate 1 (IRS1) through serine phosphorylation and thereby reduce GLUT4 translocation [40]. The resulting rise in maternal glucose availability supports concentration-dependent placental transfer through GLUT1 [41].

#### Pancreatic β-cell compensation: lactogenic and serotonergic signaling

Maternal insulin secretion rises substantially to compensate for pregnancy-associated insulin resistance [42, 43]. Lactogenic signaling through the prolactin receptor-JAK2-STAT5 pathway contributes to β-cell adaptation. In rodent models, STAT5 promotes cyclin D2 expression, β-cell expansion, and anti-apoptotic programs [42, 43]. STAT5 also induces tryptophan hydroxylase 1 and intra-islet serotonin synthesis in these models. Serotonin signaling through HTR2B then supports β-cell proliferation and functional compensation [42, 43]. Evidence for an equivalent serotonin-dependent proliferative response in human pregnancy remains limited. A case–control study associated HTR2B rs17619600 with GDM risk, but this association does not establish causality [44] (Fig. 1a).

### Cardiovascular adaptation: hemodynamic remodeling and vasodilation

Maternal plasma volume expands by approximately 40%–50% while blood pressure is generally maintained [1, 45]. A concurrent reduction in systemic vascular resistance helps accommodate this high-volume, low-pressure state [1, 45, 46].

#### The Relaxin-RXFP1-NO axis

Relaxin is secreted by the corpus luteum and later by the placenta and decidua [46, 47]. It contributes to maternal hemodynamic adaptation through RXFP1 on endothelial and vascular smooth muscle cells. RXFP1 signaling can activate Gα_i/o_-PI3K-Akt pathways and promote endothelial nitric oxide synthase (eNOS) phosphorylation [46]. Increased nitric oxide production supports vasodilation. Relaxin also increases MMP-2 and MMP-9 activity, which can process big endothelin-1 and favor endothelial ET_B_ receptor signaling [46, 47]. Renal vasodilation contributes to increased renal plasma flow and glomerular filtration during pregnancy [48] (Fig. 1b).

#### Spiral artery remodeling and trophoblast invasion

Spiral arteries are transformed from narrow, muscular vessels into low-resistance conduits during healthy placentation. Extravillous trophoblasts (EVTs) invade the decidua and myometrium through coordinated changes in adhesion and migration [49]. An integrin switch from α6β4 toward α1β1 and α5β1 accompanies the invasive phenotype [49]. EVTs also acquire vascular mimicry-like features, including VE-cadherin and VCAM-1 expression. These changes may facilitate interaction with the spiral artery endothelium. Endothelial-cell loss and smooth-muscle remodeling contribute to reduced vasoreactivity and sustained uteroplacental perfusion [49, 50] (Fig. 1b).

### Hepatic and excretory adaptation to pregnancy hormones

Pregnancy increases hepatobiliary demands through steroid metabolism and clearance of maternal and fetoplacental metabolites. Bile acid homeostasis is regulated partly by the farnesoid X receptor (FXR). FXR activation promotes ABCB11 expression and bile salt export pump (BSEP) activity [51]. It also induces small heterodimer partner, which restrains bile acid uptake and synthesis. Rising estrogen concentrations can reduce FXR-dependent transporter expression, including BSEP and MDR3 [24, 52]. Sulfated progesterone metabolites can further inhibit BSEP-mediated bile acid transport [24, 25]. These physiological pressures may approach a cholestatic threshold when hepatobiliary reserve is limited [24, 53] (Fig. 1c).

### Immunological adaptation: the tolerance paradox

Pregnancy requires maternal tolerance of fetal and placental antigens while preserving antimicrobial defense. The fetus has therefore often been described as a semi-allogeneic graft [54, 55]. Healthy gestation involves context-specific immune regulation rather than generalized immunosuppression [54–56]. Decidual natural killer cells, regulatory T-cell circuits, and specialized antigen presentation contribute to this balance. Trophoblast expression of nonclassical human leukocyte antigen molecules, particularly HLA-G, further restrains damaging alloimmune responses [56–58]. Hormonal, metabolic, and checkpoint signals integrate these mechanisms at the maternal–fetal interface.

#### The HLA-G-mediated immune regulation

EVTs lack the highly polymorphic HLA-A and HLA-B molecules but selectively express nonclassical HLA-G [59]. HLA-G forms disulfide-linked homodimers that bind inhibitory receptors on uterine natural killer (uNK) cells, including LILRB1 and KIR2DL4. These interactions support a uNK-cell phenotype oriented toward trophic and pro-angiogenic functions rather than direct cytotoxicity [59]. uNK cells produce factors such as VEGF-C, PlGF, Ang-2, and interferon-γ. These mediators support trophoblast invasion and vascular remodeling [59, 60]. HLA-G is therefore an important tolerogenic ligand at the maternal–fetal interface [61] (Fig. 1d).

#### IDO, Kynurenine, and regulatory T-Cell induction

Trophoblasts and decidual antigen-presenting cells express indoleamine 2,3-dioxygenase (IDO), which converts tryptophan to kynurenine [56]. Tryptophan depletion can limit effector T-cell proliferation and promote anergy. Kynurenine also activates the aryl hydrocarbon receptor in naïve CD4 + T cells. This signaling can favor FOXP3 expression and regulatory T-cell differentiation [56, 62] (Fig. 1d). Expanded regulatory T-cell populations support local immune tolerance through mediators including interleukin-10 (IL-10) and transforming growth factor-β (TGF-β). These effects are context-dependent and complement other decidual immune-regulatory pathways (Fig. 1d).

Healthy pregnancy therefore requires coordinated recalibration across maternal organ systems. Metabolic adaptation includes physiological insulin resistance and compensatory insulin secretion. Hemodynamic adaptation involves systemic vasodilation, increased renal filtration, and spiral artery remodeling. HLA-G signaling, decidual immune specialization, and regulatory T-cell pathways support maternal–fetal tolerance. The liver maintains bile acid homeostasis despite pregnancy-associated hormonal pressure. Within PMAFC, higher gestational/fetoplacental stress load or lower maternal adaptive reserve may shift pregnancy toward maladaptation (Fig. 2).

**Fig. 2:**
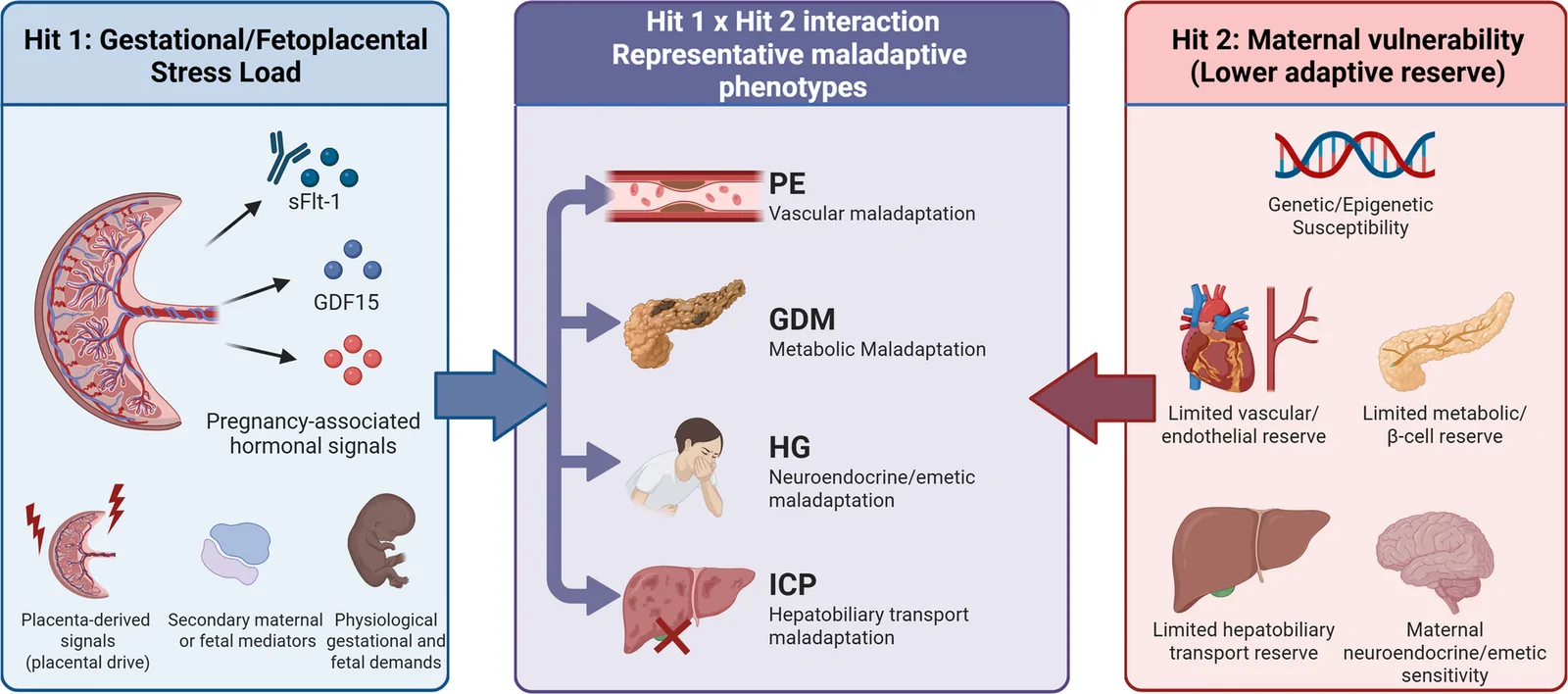
Two-hit framework for pregnancy-related disorders. Hit 1 represents the disorder-relevant gestational/fetoplacental stress load, encompassing placenta-derived signals, physiological gestational and fetal demands, and secondary maternal or fetal mediators. Placental drive denotes only the placenta-derived subset of this load. Hit 2 represents maternal vulnerability, with greater vulnerability corresponding to lower adaptive reserve. Interaction between these dimensions may shift pregnancy toward vascular, metabolic, neuroendocrine/emetic, or hepatobiliary maladaptation. PE, GDM, HG, and ICP are illustrative and potentially overlapping phenotypes rather than exhaustive or sequential categories. Hit 1 and Hit 2 are interacting conceptual dimensions, not fixed temporal stages. Abbreviations: GDF15, growth differentiation factor 15; GDM, gestational diabetes mellitus; HG, hyperemesis gravidarum; ICP, intrahepatic cholestasis of pregnancy; PE, preeclampsia; sFlt-1, soluble fms-like tyrosine kinase 1. The figures were created using BioRender.com

## Preeclampsia: failure of angiogenic and endothelial adaptation

PE provides a clinical paradigm of maternal endothelial maladaptation during pregnancy [17, 63]. Within PMAFC, it reflects an interaction between placenta-derived stress signals and maternal vascular vulnerability [17, 64]. Impaired trophoblast invasion can produce structural and hypoxic placental stress, which constitutes Hit 1. The resulting anti-angiogenic and inflammatory signals can challenge maternal endothelial reserve, which constitutes Hit 2. This section evaluates these mechanisms while distinguishing evidence from early-onset disease, experimental models, and broader PE populations (Fig. 3).

**Fig. 3:**
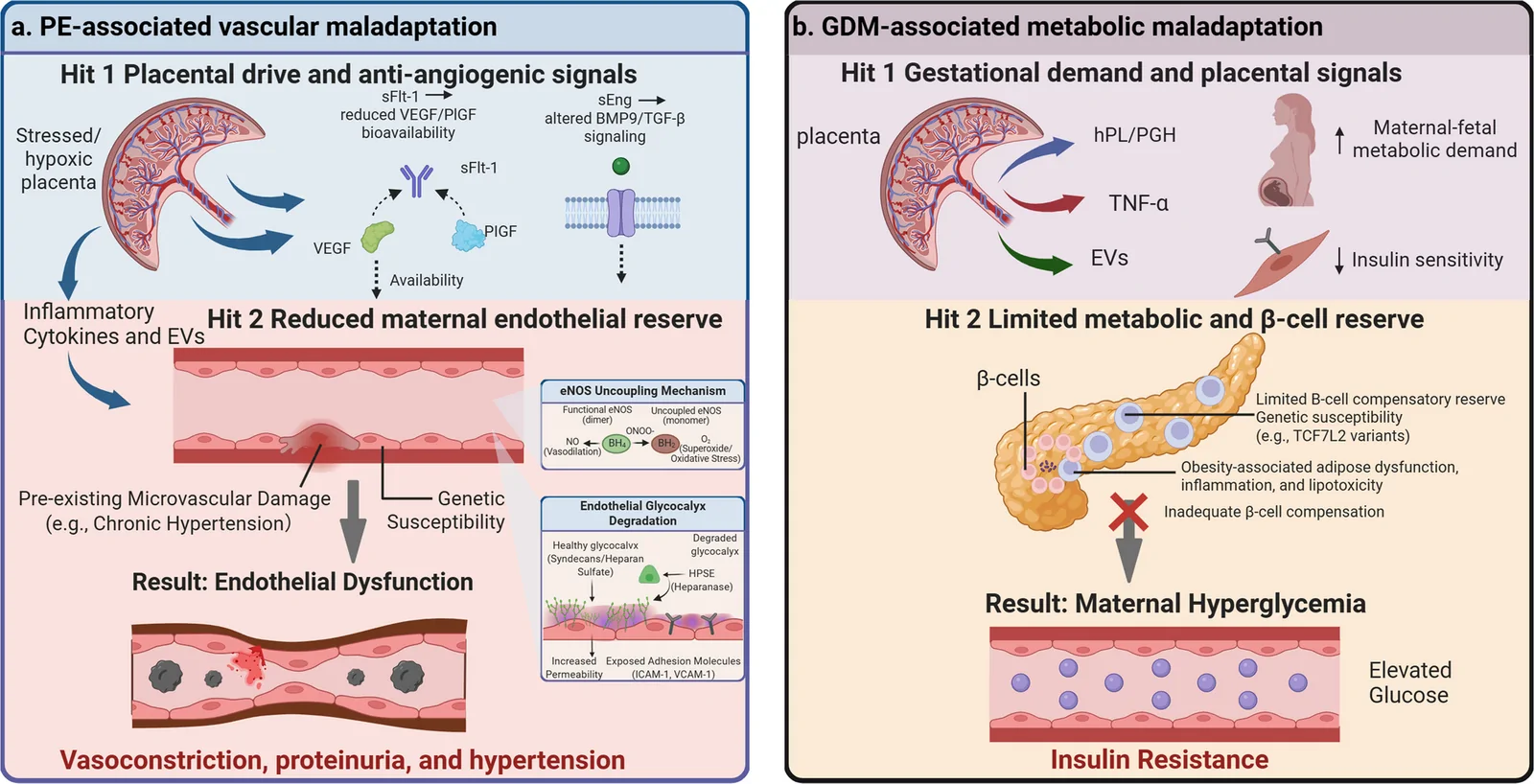
PE and GDM within the PMAFC framework. **a** In PE, placental stress, including hypoxic stress, is associated with increased anti-angiogenic signaling. sFlt-1 reduces VEGF/PlGF bioavailability, whereas sEng alters BMP9/TGF-β signaling. Together with inflammatory cytokines and EVs, these signals interact with reduced maternal endothelial reserve, including pre-existing microvascular damage and genetic susceptibility. eNOS uncoupling and endothelial glycocalyx degradation may contribute to endothelial dysfunction, vasoconstriction, proteinuria, and high blood pressure. **b** In GDM, increased maternal–fetal metabolic demand and placental signals, including hPL, PGH, TNF-α, and EVs, reduce maternal insulin sensitivity. Their interaction with limited metabolic and β-cell reserve, genetic susceptibility, and obesity-associated adipose dysfunction, inflammation, and lipotoxicity may result in inadequate β-cell compensation, and maternal hyperglycemia. The pathways shown are representative and heterogeneous, and individual pregnancies may involve only a subset of these mechanisms. Abbreviations: BH4, tetrahydrobiopterin; BMP9, bone morphogenetic protein 9; eNOS, endothelial nitric oxide synthase; EVs, extracellular vesicles; GDM, gestational diabetes mellitus; hPL, human placental lactogen; HPSE, heparanase; ICAM-1, intercellular adhesion molecule 1; NO, nitric oxide; PE, preeclampsia; PGH, placental growth hormone; PlGF, placental growth factor; PMAFC, Placental-Maternal Adaptation Failure Continuum; sEng, soluble endoglin; sFlt-1, soluble fms-like tyrosine kinase 1; TCF7L2, transcription factor 7-like 2; TGF-β, transforming growth factor-β; TNF-α, tumour necrosis factor-α; VCAM-1, vascular cell adhesion molecule 1; VEGF, vascular endothelial growth factor. The figures were created using BioRender.com

### Hit 1: Placental drive and anti-angiogenic signaling

In early-onset PE, especially before 34 weeks, Hit 1 is often dominated by impaired placentation and placenta-derived stress. Intermittent perfusion, hypoxic stress, and release of anti-angiogenic or inflammatory factors contribute to the maternal syndrome [17, 65].

#### Impaired deep placentation: integrins and notch signaling

Normal spiral artery remodeling requires EVT differentiation and invasion of the decidua and inner myometrium. In PE, EVTs may retain α6β4 and show an incomplete switch toward the invasive α1β1 phenotype [66]. Reduced invasion leaves spiral arteries incompletely remodeled and preserves vasoreactive smooth muscle [67]. Single-cell studies have also identified altered trophoblast differentiation programs in PE placentas [68]. Notch2 signaling may contribute to perivascular trophoblast differentiation, although its role in human PE requires further validation [68, 69]. Reduced uterine corin expression provides another potential mechanism because corin activates atrial natriuretic peptide locally [70]. Evidence from corin-deficient mice supports biological plausibility but does not establish a universal human pathway.

#### Hypoxia-inducible factor signaling

Incomplete placentation can produce intermittent perfusion and ischemia–reperfusion stress. Reactive oxygen species may inhibit prolyl hydroxylases and thereby reduce von Hippel-Lindau-mediated degradation of hypoxia-inducible factors (HIFs) [71–74]. Stabilized HIFs then activate hypoxia-responsive genes. In trophoblasts, HIF-2α appears particularly important for hypoxia-induced Fms-related receptor tyrosine kinase 1 (FLT1) expression [75]. HIF-1α may contribute to broader stress responses, but the relative roles of HIF isoforms vary by cell type and experimental model [72, 75].

#### Anti-angiogenic signaling: sFlt-1 and soluble endoglin

Increased soluble anti-angiogenic factors are central features of PE. sFlt-1 sequesters VEGF and PlGF, thereby reducing endothelial pro-angiogenic signaling [76, 77]. Alternative FLT1 splicing produces placenta-enriched sFlt-1 isoforms, including sFlt-1-e15a [78, 79]. Altered JMJD6 and U2AF65 activity may favor this splice pattern in PE placentas [78, 79]. Oxidative stress can also promote shedding of soluble endoglin, which interferes with BMP9 and TGF-β signaling [80]. Experimental elevation of sFlt-1 reproduces hypertension and proteinuria, whereas additional soluble endoglin can intensify hepatic and hematological injury [81, 82]. These models support causality for selected pathways but do not capture the full heterogeneity of human PE [82, 83] (Fig. 3a).

#### Syncytiotrophoblast extracellular vesicles and damage signals

Stressed syncytiotrophoblasts release extracellular vesicles (EVs) and trophoblastic debris into the maternal circulation [84, 85]. These products can carry damage-associated molecular patterns, including high-mobility group box 1 (HMGB1) and cell-free fetal DNA [19, 86]. HMGB1 can activate Toll-like receptor 4 (TLR4) and promote inflammatory cytokine release [87, 88]. Cell-free fetal DNA is more consistently linked to nucleic-acid sensors such as TLR9 [89, 90]. Syncytiotrophoblast EVs also alter monocyte and macrophage responses. However, the direction of these effects varies by PE phenotype, vesicle preparation, and experimental system [85, 91].

### Hit 2: reduced maternal endothelial reserve

The maternal endothelium is a major target of placenta-derived stress. Clinical disease is not determined by Hit 1 alone, because endothelial reserve and pre-existing vascular risk modify the response [17, 64, 92].

#### Endothelial dysfunction and eNOS uncoupling

VEGF and PlGF support endothelial nitric oxide production during healthy pregnancy. In PE, reduced angiogenic-factor bioavailability can impair this pathway, particularly when chronic hypertension or obesity has already reduced vascular reserve. Functional endothelial nitric oxide synthase (eNOS) requires tetrahydrobiopterin (BH4) to couple electron transfer to nitric oxide synthesis. Oxidation of BH4 to BH2 can uncouple eNOS and redirect electrons toward superoxide production [93, 94]. Superoxide further reduces nitric oxide bioavailability and amplifies oxidative stress. This cycle can contribute to vasoconstriction, hypertension, and platelet activation, although multiple endothelial pathways participate in the clinical syndrome (Fig. 3a).

#### Endothelial glycocalyx injury

The endothelial glycocalyx is a proteoglycan- and glycosaminoglycan-rich surface layer that regulates permeability and leukocyte adhesion [95, 96]. Glycocalyx degradation has been reported in PE, particularly in early-onset disease [97, 98]. Heparanase cleaves heparan sulfate and can promote loss of glycocalyx integrity [99]. Hypoxic trophoblast systems also show heparanase-dependent regulation of sFlt-1 release [100]. These observations support a possible link between placental stress and maternal surface-layer injury. Glycocalyx loss may expose adhesion molecules and increase vascular permeability [95, 96], but direct causal evidence in human PE remains limited [97–100] (Fig. 3a).

#### Podocyte injury and proteinuria

Podocyte-derived VEGF supports the glomerular filtration barrier and slit-diaphragm integrity [101, 102]. Excess sFlt-1 can reduce local VEGF availability and disrupt endothelial-podocyte signaling in PE [82, 103, 104]. Associated changes include reduced nephrin and synaptopodin expression, cytoskeletal rearrangement, and foot-process effacement [102–104]. Glomerular endotheliosis and proteinuria often improve after delivery as placental anti-angiogenic drive declines. Recovery may nevertheless take weeks or months, and some women retain increased long-term renal risk [103–105].

#### Endoplasmic reticulum stress and the unfolded protein response

Oxidative stress and protein misfolding can activate PERK, IRE1α, and ATF6 in endothelial and placental cells [106, 107]. The unfolded protein response initially reduces translational load and promotes cellular recovery. Persistent stress can instead increase C/EBP homologous protein and pro-apoptotic signaling [106–108]. Increased circulating endothelial cells in PE are consistent with endothelial injury and detachment [109, 110]. However, these markers are not specific to endoplasmic reticulum stress, and their causal contribution varies across experimental systems.

PE therefore illustrates a two-hit interaction between placenta-derived stress and maternal vascular vulnerability [17]. sFlt-1, soluble endoglin, EVs, and inflammatory mediators constitute important components of Hit 1. Endothelial repair capacity, nitric oxide bioavailability, and pre-existing vascular disease influence Hit 2 [17, 92, 94]. Postpartum risk is more plausibly linked to broad epigenetic remodeling and maladaptive vascular repair than to a single persistent molecular lesion. Candidate changes include altered methylation, vascular stiffness, fibrosis, and inflammatory activation [111, 112].

## Gestational diabetes mellitus: failure of metabolic adaptation

GDM develops when pregnancy-associated insulin resistance exceeds maternal compensatory capacity. Placental hormones and broader gestational demands normally facilitate fetal nutrient supply [42, 43]. GDM emerges when β-cell compensation is inadequate, often in the context of genetic, epigenetic, or metabolic susceptibility [16, 42, 43, 113]. Placental endocrine and inflammatory signaling may amplify maternal insulin resistance [20, 21]. Placenta-derived EVs may provide an additional communication pathway [114, 115]. Within PMAFC, these signals form part of the wider gestational/fetoplacental stress load rather than an exclusive placental cause of GDM (Fig. 3b).

### Hit 1: gestational metabolic stress and placental signaling

Placental endocrine, inflammatory, and extracellular-vesicle signals can amplify the physiological insulin resistance of pregnancy [20]. They should be interpreted as components of a broader metabolic stress load that also includes maternal adiposity, nutrient flux, and fetal demand.

#### Placenta-derived extracellular vesicles and MicroRNA cargo

Placenta-derived EVs provide a route for communication with maternal liver, muscle, adipose tissue, and pancreatic islets [114, 116]. Human studies have associated GDM with altered concentrations and microRNA profiles of circulating placental EVs [115]. Functional experiments showed that vesicles from GDM pregnancies can impair insulin-stimulated glucose uptake in skeletal muscle cells [115]. Reviews also implicate microRNA cargo in regulation of PI3K-Akt-GLUT4 signaling [114, 117]. A recent study reported that exosomal miR-320b can worsen β-cell dysfunction in GDM models [118]. These findings support biological plausibility but do not establish EVs as necessary or sufficient causes of human GDM (Fig. 3b).

#### Placental inflammation, TNF-α, and adipokines

GDM placentas show altered inflammatory-cell profiles and cytokine signaling, although findings vary across studies [119, 120]. Placental and maternal TNF-α can activate JNK and promote inhibitory serine phosphorylation of IRS1 [121]. This change reduces IRS-PI3K-Akt signaling and may worsen peripheral insulin resistance. Increased placental leptin expression has also been reported in GDM [122]. Impaired leptin responsiveness may favor lipid accumulation and metabolic dysfunction. These signals interact with, rather than replace, maternal adipose-tissue inflammation and lipotoxicity [123–126] (Fig. 3b).

### Hit 2: limited β-cell reserve and adipose-tissue dysfunction

Within PMAFC, Hit 2 represents limited maternal metabolic reserve, particularly inadequate β-cell compensation and obesity-associated adipose dysfunction.

#### β-cell dysfunction and dedifferentiation-like changes

β-Cell dedifferentiation is established mainly in experimental diabetes models, not as a defining feature of human GDM [127, 128]. Loss of FoxO1-dependent identity programs reduces MafA, PDX1, and NKX6.1 expression in these models. Progenitor-associated markers can re-emerge as insulin secretion declines [127, 128]. Hyperglycemia, lipotoxicity, and oxidative stress could favor similar changes during GDM. Direct longitudinal evidence from human pregnancy, however, remains limited. Islet amyloid polypeptide can activate NLRP3-related signaling [129], but its specific contribution to human GDM is uncertain (Fig. 3b).

#### Genetic susceptibility

Genome-wide analyses show substantial overlap between the genetic architectures of GDM and type 2 diabetes mellitus [130]. Transcription factor 7-like 2 (TCF7L2) variants are consistently associated with GDM risk and may impair incretin-coupled insulin secretion [113, 131]. Pathogenic GCK variants reset the glucose-sensing threshold and can produce fasting hyperglycemia [132]. MTNR1B variants link circadian regulation to insulin secretion and GDM susceptibility [133, 134]. Observational data also associate irregular or evening shift work with GDM risk [135]. These findings support multifactorial susceptibility but do not imply that a single variant determines disease expression (Fig. 3b).

#### Adipose-tissue dysfunction and metabolic inflammation

Obesity-associated adipose expansion can produce local hypoxia, adipocyte stress, and macrophage recruitment [123, 124]. Crown-like structures reflect macrophage accumulation around damaged adipocytes. This microenvironment increases mediators such as IL-6 and TNF-α, which can amplify systemic insulin resistance. Lipid intermediates provide a parallel mechanism. Ceramides can activate protein phosphatase 2A and reduce Akt phosphorylation, thereby weakening insulin signaling [125]. These pathways may interact with placental inflammation but are not specific to pregnancy.

### A proposed fetal-placental feed-forward mechanism

The revised Pedersen hypothesis links maternal hyperglycemia to fetal hyperglycemia and compensatory fetal hyperinsulinemia [136, 137]. Human placental studies associate increased insulin or IGF-I-mTORC1 signaling with amino acid transport and fetal overgrowth [138, 139]. These findings support enhanced placental nutrient sensing in GDM and maternal obesity. They do not establish that fetal insulin directly activates placental mTORC1 in humans. Nor do they confirm a self-amplifying fetoplacental loop. PMAFC therefore treats placental nutrient transport and fetal hyperinsulinemia as potential metabolic stress contributors, not as a proven feedback mechanism.

GDM reflects interactions between gestational metabolic demand and limited maternal adaptive capacity. Inadequate β-cell compensation is a central component of maternal vulnerability [42, 43]. Adipose inflammation and lipotoxicity can further increase insulin resistance [123–125]. Maternal hyperglycemia and fetal hyperinsulinemia then contribute to fetal overgrowth [136, 137]. Postpartum metabolic consequences and surveillance are discussed in Sects. "Pregnancy memory: postpartum molecular, vascular, and metabolic imprints" and "Clinical management and translational strategies".

## Hyperemesis gravidarum and intrahepatic cholestasis of pregnancy: neuroendocrine and hepatobiliary transport maladaptation

Pregnancy also challenges maternal neuroendocrine signaling and hepatobiliary transport. HG and ICP illustrate disorder-specific forms of gestational/fetoplacental stress load. In HG, Hit 1 is dominated by fetoplacental GDF15 exposure, whereas Hit 2 reflects maternal sensitivity to emetic signaling. In ICP, Hit 1 comprises gestational hormonal pressure on transporter expression and function. Hit 2 reflects hepatobiliary vulnerability, including reduced ABCB11- or ABCB4-related transport capacity. Circulating bile acids arise downstream of impaired transport and can act as disease markers and context-dependent mediators [22, 140–142] (Fig. 4).

**Fig. 4:**
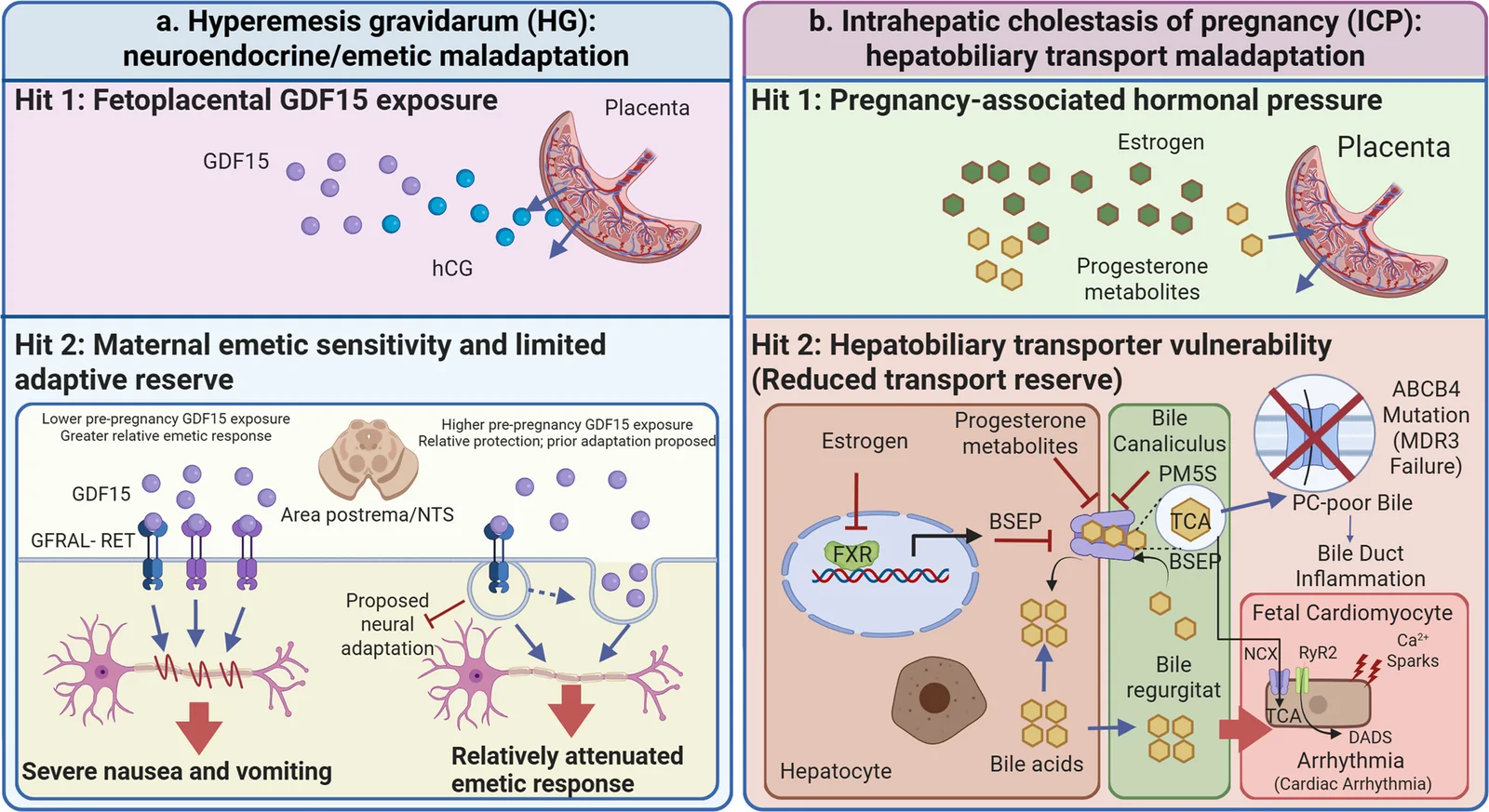
Hyperemesis gravidarum and intrahepatic cholestasis of pregnancy within the Placental-Maternal Adaptation Failure Continuum framework. The schematic presents neuroendocrine/emetic and hepatobiliary transport maladaptation as illustrative examples of gestational/fetoplacental stress load interacting with maternal adaptive reserve. **a** In HG, Hit 1 is dominated by the pregnancy-related rise in fetoplacental GDF15; hCG is shown only as a correlated gestational signal. Hit 2 reflects maternal neuroendocrine/emetic vulnerability, including variation in adaptation to GDF15-GFRAL-RET signaling in the area postrema and nucleus tractus solitarius. Lower pre-pregnancy GDF15 exposure may provide less prior adaptation to the gestational surge and increase susceptibility to severe nausea and vomiting. Higher prior exposure may attenuate the emetic response, although receptor desensitization remains a proposed rather than directly established human mechanism. **b** In ICP, Hit 1 comprises pregnancy-associated hormonal pressure on hepatobiliary transport. Estrogen receptor signaling may reduce FXR-RXR-dependent ABCB11 transcription, whereas sulfated progesterone metabolites, including PM5S, can inhibit BSEP-mediated bile acid transport. Hit 2 reflects limited canalicular transport reserve, illustrated here by ABCB4/MDR3 dysfunction. BSEP inhibition may promote bile acid accumulation and regurgitation, whereas ABCB4/MDR3 dysfunction may reduce phosphatidylcholine secretion, produce phosphatidylcholine-poor bile, and contribute to cholangiocyte injury and cholestatic inflammation. Severe ICP is associated with fetal cardiac dysfunction. Experimental cardiomyocyte studies support a possible taurocholate-related arrhythmogenic mechanism involving altered calcium handling, but this has not been established as the principal cause of fetal death in human ICP. Abbreviations: BSEP, bile salt export pump; FXR, farnesoid X receptor; GDF15, growth differentiation factor 15; GFRAL, GDNF family receptor alpha-like; hCG, human chorionic gonadotropin; MDR3, multidrug resistance protein 3; PM5S, epiallopregnanolone sulfate; RET, rearranged during transfection; RXR, retinoid X receptor; ABCB4, ATP-binding cassette subfamily B member 4; NTS, nucleus tractus solitarius; DADs, delayed afterdepolarizations. The figures were created using BioRender.com

### Hyperemesis gravidarum: GDF15-GFRAL signaling and emetic adaptation

HG was historically attributed to psychogenic factors or human chorionic gonadotropin alone. Genomic and endocrine evidence now supports a major role for the GDF15-GFRAL axis [22, 143]. Fetoplacental GDF15 exposure interacts with maternal emetic sensitivity, providing a mechanistic explanation for severe nausea and vomiting in susceptible pregnancies [22, 143, 144].

#### Hit 1: fetoplacental GDF15 exposure

GDF15 is a stress-responsive member of the TGF-β superfamily [145, 146]. During pregnancy, the fetoplacental unit becomes a major source of circulating GDF15 [22, 140]. Maternal concentrations rise across gestation and are generally higher in women with HG than in unaffected controls [22, 147–149]. GDF15 and human chorionic gonadotropin show overlapping early-pregnancy patterns, but human chorionic gonadotropin alone does not explain HG [148–150]. Twin gestation and gestational trophoblastic disease can increase fetoplacental signal exposure and are associated with HG risk [22, 148]. These associations support a high-exposure component without defining a universal concentration threshold (Fig. 4a).

#### Hit 2: pre-pregnancy exposure and GFRAL-RET signaling

GDF15 binds GFRAL, whose expression is concentrated in the area postrema and nucleus tractus solitarius [145, 151]. GFRAL recruits the RET co-receptor and activates neuronal pathways that regulate nausea, vomiting, and food intake. Variation in symptoms despite high gestational GDF15 may partly reflect differences in pre-pregnancy exposure and neural adaptation. A recent human genetic study associated higher pre-pregnancy GDF15 exposure with relative protection from HG [22]. Lower baseline exposure may therefore produce a larger relative change when placental GDF15 rises. Receptor desensitization is a plausible interpretation, not a directly established human mechanism. IGFBP7 has also been genetically associated with HG, but its receptor biology and causal contribution remain uncertain [143, 148] (Fig. 4a).

### Intrahepatic cholestasis of pregnancy: a hepatobiliary transport bottleneck

ICP can be viewed as a gene-environment interaction [152, 153]. Pregnancy-associated hormonal changes challenge bile acid transport [153], while genetic variation in ABCB11, ABCB4, and related genes modifies maternal susceptibility [153–155].

#### Hit 1: hormonal effects on canalicular transport

Bile salt export pump (BSEP), encoded by ABCB11, mediates the rate-limiting step in canalicular bile acid export [156]. Estrogen receptor signaling can interfere with FXR-RXR-dependent ABCB11 transcription and reduce transport capacity [23, 24, 141]. Sulfated progesterone metabolites provide a second challenge by inhibiting BSEP-mediated bile acid transport [157, 158]. Structural studies of human BSEP show how small molecules can restrict transporter conformational cycling [159]. These data provide a mechanistic framework but do not establish the exact binding mode of every pregnancy-associated metabolite. Together, reduced expression and functional inhibition can expose limited hepatobiliary reserve (Fig. 4b).

#### Hit 2: ABCB11- and ABCB4-related susceptibility

ABCB11 variants can reduce BSEP expression, trafficking, or transport activity. The common V444A variant is associated with ICP susceptibility and may lower residual export capacity [152, 156]. Selected missense variants, including E297G, impair protein processing and canalicular localization in experimental systems [160]. ABCB4 variants affect MDR3-mediated phosphatidylcholine secretion and create a distinct vulnerability. Reduced phosphatidylcholine leaves bile acids less effectively buffered within mixed micelles. This can increase cholangiocyte membrane injury and cholestatic inflammation [152, 154, 155]. Genetic variants explain only a subset of ICP and should not be treated as universal causes (Fig. 4b).

#### Bile acid-mediated mechanisms of fetal risk

Sudden intrauterine fetal death is the most serious complication of ICP, but its mechanism remains incompletely understood [161, 162]. Higher maternal bile acid concentrations are associated with fetal cardiac dysfunction [163, 164]. Taurocholate impairs contractility and calcium handling in experimental rat cardiomyocytes [165, 166]. These observations support the plausibility of an arrhythmogenic pathway. They do not establish arrhythmia as the sole or primary cause of fetal death in human ICP. Conventional surveillance may not predict every acute event, but this limitation should not be attributed to one mechanism alone [161, 162] (Fig. 4b).

HG and ICP show how pregnancy can reveal latent receptor-level or transporter-related vulnerability. In HG, pre-pregnancy GDF15 exposure may modify the response to gestational signals [22]. In ICP, transporter variation can limit the response to hormonal and bile acid stress [141, 152]. These pathways may eventually support more precise risk stratification. Their clinical value, however, requires prospective validation and pregnancy-specific safety evidence [22, 141, 156].

## A unified framework: the placental-maternal adaptation failure continuum

Conventional classifications separate vascular, metabolic, emetic, and hepatobiliary pregnancy disorders. This approach can obscure selected areas of mechanistic overlap without implying that the disorders share one pathophysiology [3, 17, 22, 167]. PMAFC offers an organizing perspective based on interactions between gestational/fetoplacental stress load and maternal adaptive reserve. It places heterogeneous PRD phenotypes on a conceptual landscape rather than a fixed biological sequence [17, 167–169]. PMAFC is therefore a heuristic framework, not a proven causal law or validated clinical model (Fig. 5).

**Fig. 5:**
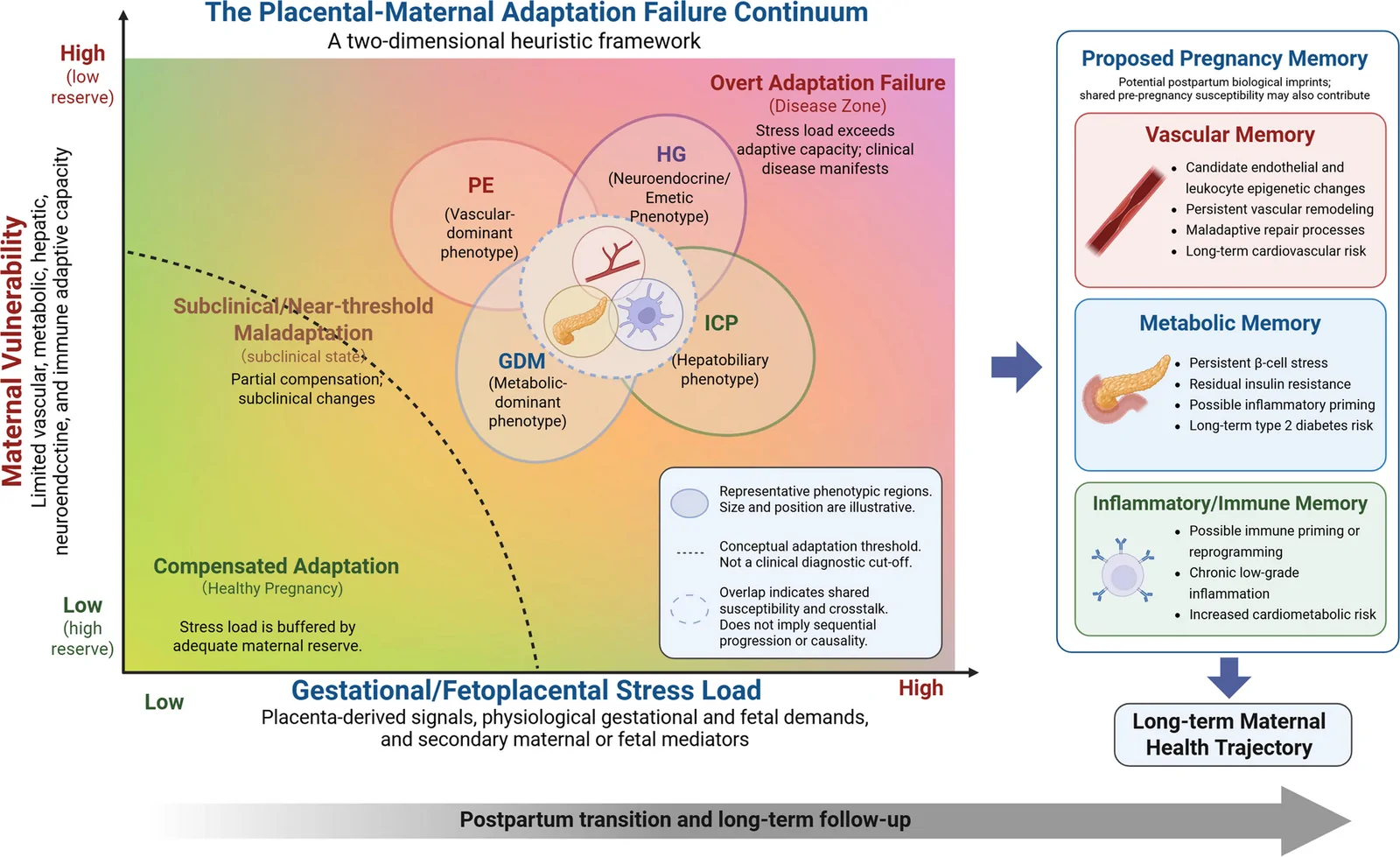
Placental-maternal adaptation failure continuum (PMAFC) and proposed pregnancy memory. The x-axis denotes increasing gestational/fetoplacental stress load, comprising placenta-derived signals, physiological gestational and fetal demands, and secondary maternal or fetal mediators. The y-axis denotes increasing maternal vulnerability, represented as the inverse of adaptive reserve. The dashed curve represents a conceptual adaptation threshold between compensated adaptation, near-threshold or subclinical maladaptation, and overt adaptation failure; it is not a diagnostic or prognostic cut-off. The size, position, and overlap of the PE, GDM, HG, and ICP phenotypic regions are illustrative. Overlap indicates shared susceptibility and crosstalk, not sequential progression or causality. The right panel depicts proposed postpartum vascular, metabolic, and inflammatory/immune imprints, including candidate epigenetic changes. Blue arrows indicate proposed links between pregnancy-related maladaptation, postpartum biological imprints, and long-term maternal health, rather than established causal pathways. Long-term cardiometabolic risk may reflect pre-existing susceptibility, shared risk factors, and maladaptation-associated biological reprogramming. Abbreviations: GDM, gestational diabetes mellitus; HG, hyperemesis gravidarum; ICP, intrahepatic cholestasis of pregnancy; PE, preeclampsia. The figures were created using BioRender.com

### Gestational/fetoplacental stress load, maternal adaptive reserve, and the adaptation threshold

Building on systems-level and two-stage models of obstetric disease, PMAFC uses two conceptual dimensions [3, 12, 17]. The first is gestational/fetoplacental stress load, defined as the total adaptive challenge imposed during pregnancy. It includes directly placenta-derived signals, physiological gestational and fetal demands, and secondary maternal or fetal mediators. Placental drive is the narrower subset produced or released by the placenta.

The second dimension is maternal adaptive reserve. It represents integrated buffering, compensation, repair, and recovery across vascular, metabolic, hepatic, neuroendocrine, and immune systems. Maternal vulnerability is defined as the inverse of adaptive reserve for graphical purposes. Higher vulnerability therefore denotes lower reserve.

This distinction is especially important in ICP. Circulating bile acids are downstream maternal metabolites and biomarkers of established cholestasis [6, 23]. They are neither placenta-derived signals nor direct measures of maternal adaptive reserve. Previous ICP, hepatobiliary disease, limited transporter capacity, and ABCB11 or ABCB4 variants are more appropriate susceptibility markers [23, 170]. Altered bile acid profiles may also affect fetal or metabolic signaling, but evidence varies by context and study design [26, 27].

Neither gestational/fetoplacental stress load nor maternal adaptive reserve can be represented by one universal biomarker. Candidate measures should be classified by biological role, intended use, and timing. Relevant categories include antecedent susceptibility markers, dynamic research measures of reserve, biomarkers of established disease activity, and indicators of organ injury.

Perfused boundary region and carotid-femoral pulse-wave velocity may provide research-level information on vascular phenotype before disease onset [97, 171]. After onset, the same measures are more likely to reflect established dysfunction. Oral glucose tolerance testing and circulating bile acids primarily indicate disease activity [6, 23, 172]. Liver enzyme abnormalities indicate biochemical organ involvement rather than reserve. Table 1 organizes these measures by biological role and assessment window.

**Table 1:** Operational categories, assessment windows, and proposed roles of candidate measures within the PMAFC framework

Domain	Antecedent susceptibility markers	Dynamic adaptive-reserve measures	Intended assessment window	Disease activity or diagnostic markers	Established organ-injury markers	Evidence level and proposed use
Vascular	• Chronic hypertension • Obesity • Chronic kidney disease • Previous PE	• PBR • cf-PWV • Cardiac output • Longitudinal blood-pressure trajectory	**Preconception/booking:** baseline risk **During pregnancy:** research vascular phenotyping; predictive utility remains unvalidated **Mid- to late pregnancy:** dynamic vascular phenotyping **Suspected PE:** clinical risk stratification	• sFlt-1/PlGF ratio (gestational-age- and assay-dependent) • Blood pressure	• Proteinuria • Serum creatinine • Platelet count • ALT/AST	Baseline risk factors and PE clinical markers are established [173]. The sFlt-1/PlGF ratio is an adjunct in selected pathways [174] PBR, cf-PWV, and cardiac output are candidate research phenotyping measures, not independent PE diagnostic or delivery-decision tools. [97, 171, 175]
Metabolic	• BMI/obesity • Previous GDM • Family history of diabetes • TCF7L2 and MTNR1B variants	• Fasting insulin • HOMA-IR • Disposition index	**Preconception/booking:** baseline risk **Early pregnancy:** investigational phenotyping **24–28 weeks:** diagnostic OGTT **After diagnosis:** serial fasting/postprandial glucose	**Diagnostic:** OGTT at 24–28 weeks **Disease control:** fasting and postprandial glucose	*No single established organ-injury marker specific to GDM* • Assess hypertensive, hepatic, renal, fetal-growth, and neonatal complications using standard clinical measures	Clinical risk factors, OGTT, and glucose monitoring are established, whereas OGTT is diagnostic rather than a direct measure of reserve [172, 176] Fasting insulin, HOMA-IR, and disposition index are mainly research measures [42]. OGTT is diagnostic, not a direct measure of reserve. [113, 133]
Hepatic	• Previous ICP • Underlying or latent liver disease • ABCB11/ABCB4 variants	*No validated routine clinical measure of hepatic adaptive reserve*	**Preconception/booking:** antecedent risk and liver history **Pregnancy:** symptom-triggered assessment **Suspected/diagnosed ICP:** serial biochemistry	• Total bile acids	• ALT • AST • GGT • Bilirubin	Previous ICP, hepatobiliary disease, and transporter variants indicate susceptibility [23, 170] Total bile acids indicate disease activity/risk, not reserve. ALT, AST, GGT, and bilirubin reflect hepatobiliary biochemical abnormality, not direct fetal risk. [6, 161]
Neuroendocrine	• Previous HG • Pre-pregnancy GDF15 exposure or baseline GDF15-related susceptibility • GDF15/IGFBP7- and related pathway variants	*No validated routine clinical measure of neuroendocrine adaptive reserve*	**Preconception/early pregnancy:** susceptibility (research) **Symptom onset, usually first trimester:** severity assessment **Active HG:** serial symptoms, weight, hydration, and electrolytes	• PUQE score • Weight loss • Dehydration • Electrolyte abnormalities	*No specific established organ-injury biomarker* • Assess renal, hepatic, neurologic, and nutritional complications as clinically indicated	Previous HG and clinical severity measures are clinically relevant. PUQE, weight loss, dehydration, and electrolyte abnormalities indicate severity or complications, not reserve [177] Pre-pregnancy GDF15 exposure and GDF15-, IGFBP7-, and RYR2-related susceptibility markers remain investigational [22, 143, 178]
Immune-inflammatory	• Baseline inflammatory profiles • Epigenetic or trained-immunity-like features (research only)	Investigational inflammatory phenotyping measures: CRP, IL-6, and NLRP3-related measures	**Preconception/early pregnancy:** research baseline **Longitudinal pregnancy sampling:** research phenotyping **Clinical use:** adjunctive and context-specific only	*No validated domain-specific disease-activity or diagnostic marker*	*No domain-specific established organ-injury marker* • Use disease-specific clinical and laboratory markers to assess organ injury	Baseline inflammatory, epigenetic, and trained-immunity-like profiles remain investigational [28, 29]. Inflammatory and NLRP3-related measures are non-specific research tools affected by infection, obesity, and other inflammatory states [9, 19, 179]. They should not be used independently for diagnosis, prognosis, treatment, or delivery decisions

Antecedent susceptibility markers indicate pre-existing or early-pregnancy risk and are conceptually distinct from dynamic adaptive-reserve measures. Disease-activity/diagnostic markers and organ-injury markers should not be interpreted as direct measures of maternal reserve. PBR and cf-PWV may be studied for research vascular phenotyping during pregnancy, but their predictive utility remains unvalidated; neither is an independent diagnostic test for PE. OGTT is a diagnostic test for GDM and not a direct measure of metabolic reserve. No validated routine clinical reserve measure is currently available for the hepatic, neuroendocrine, or immune-inflammatory domains

*ALT* alanine aminotransferase, *AST* aspartate aminotransferase, *BMI* body mass index, *cf-PWV* carotid-femoral pulse wave velocity, *CKD* chronic kidney disease, *CRP* C-reactive protein, *GDM* gestational diabetes mellitus, *GGT* gamma-glutamyl transferase, *HG* hyperemesis gravidarum, *HOMA-IR* homeostatic model assessment of insulin resistance, *ICP* intrahepatic cholestasis of pregnancy, *IL-6* interleukin-6, *OGTT* oral glucose tolerance test, *PBR* perfused boundary region, *PE* preeclampsia, *PMAFC* Placental-Maternal Adaptation Failure Continuum, *PUQE* Pregnancy-Unique Quantification of Emesis and Nausea, *sFlt-1/PlGF* soluble fms-like tyrosine kinase-1/placental growth factor

The adaptation threshold is the conceptual point at which stress load exceeds available adaptive capacity. It is not a validated diagnostic, prognostic, or therapeutic cutoff. Its position can change across individuals and gestation because both dimensions are dynamic. Hit 1 and Hit 2 are likewise not fixed temporal stages. Maternal vulnerability may predate conception, while gestational signals can act as triggers, amplifiers, or unmasking events.

High maternal vulnerability may allow modest stress to produce maladaptation. Conversely, a high stress load may exceed initially adequate reserve. A proposed near-threshold zone lies between compensated adaptation and categorical disease. Transient or borderline abnormalities may occur within this zone. It is a hypothesis-generating representation of graded biological risk, not a new diagnosis. A single abnormal oral glucose tolerance test value illustrates graded metabolic risk because it predicts later type 2 diabetes [172, 180].

The relative contributions of stress load and vulnerability differ among disorders. PE often involves placenta-derived angiogenic and inflammatory stress interacting with maternal vascular vulnerability [17–19]. GDM combines gestational metabolic demand with limited β-cell and metabolic reserve [20, 21, 42]. HG combines fetoplacental GDF15 exposure with maternal neuroendocrine sensitivity [22]. ICP combines gestational hormonal stress with transporter-related hepatobiliary vulnerability [23–25, 170]. These mappings organize heterogeneous phenotypes but are neither mutually exclusive nor quantitatively validated.

### Shared stress-response networks and cross-disorder associations

Selected PRDs may involve overlapping vascular, metabolic, hepatic, neuroendocrine, and immune-inflammatory networks [3, 9, 17]. Associations among HG, ICP, GDM, and PE may reflect shared susceptibility, common gestational stressors, bidirectional crosstalk, or residual confounding. They should therefore be interpreted as context-dependent and hypothesis-generating. They do not establish fixed temporal progression from one disorder to another (Fig. 5).

#### Hyperemesis gravidarum as a potential threshold-modifying phenotype

Within PMAFC, HG may modify the adaptation threshold but is not a proven precursor of other PRDs. Fetoplacental GDF15 exposure and maternal emetic sensitivity define its main neuroendocrine components [22, 143]. Persistent vomiting, dehydration, nutritional depletion, ketosis, and aminotransferase elevation can add secondary metabolic and hepatic stress [5, 181, 182]. These consequences may reduce the available reserve margin. Observational studies link severe or persistent HG to selected placenta-mediated outcomes, but associations with later GDM are heterogeneous [183–185]. One cohort also reported higher bile acids among ICP cases with first-trimester HG [186]. Shared predisposition, residual confounding, and consequences of severe illness remain alternative explanations. HG should therefore be treated as a possible sensitizing state rather than a deterministic cause of ICP, GDM, or PE.

#### Intrahepatic cholestasis as a context-dependent systemic modifier

ICP reflects gestational hormonal stress interacting with maternal hepatobiliary vulnerability [23, 25, 152, 170]. Circulating bile acids are downstream metabolites and disease-activity biomarkers [6, 23]. They are not components of placental drive or direct measures of reserve. This distinction prevents established cholestasis from being misclassified as an antecedent vulnerability.

Bile acids signal through receptors including FXR and TGR5. Altered profiles are associated with disturbed glucose homeostasis in experimental and observational studies [26, 27, 187, 188]. Severe ICP has also been associated with maternal endothelial dysfunction in one case–control study [189]. Direct endothelial toxicity or systemic amplification in human ICP, however, remains insufficiently established. Associations of ICP with GDM and PE do not demonstrate causality [188, 190]. Its proposed amplifying role should therefore remain context-dependent and hypothesis-generating.

#### Shared vascular-metabolic-immune stress networks

Oxidative stress, impaired nitric oxide signaling, inflammasome activation, and metabolic inflammation may converge across selected PRDs [3, 9]. Endothelial dysfunction provides one area of overlap between PE and GDM [9]. Altered placental and maternal sFlt1/PlGF expression has also been reported in GDM [191]. Its clinical significance remains uncertain. eNOS dysregulation and NLRP3-related signaling offer plausible links between oxidative, inflammatory, and metabolic stress [19, 179, 192, 193]. Hyperglycemia, altered bile acid signaling, EVs, and cytokines may enter these networks through distinct upstream routes [26, 27, 85, 114]. PMAFC treats this convergence as a modifier of adaptation, not evidence for an obligatory disease sequence.

### Pregnancy memory: postpartum molecular, vascular, and metabolic imprints

Pregnancy memory denotes persistent molecular, cellular, or structural imprints after a complicated pregnancy. This concept does not imply that pregnancy complications alone cause later chronic disease. Long-term clinical risk after pregnancy complications is supported by cardiovascular outcome studies [194–196]. Persistent immune, epigenetic, and vascular changes have also been reported [28–31, 197], although these findings do not exclude pre-existing susceptibility or shared risk factors. Weight-cycling data provide only a non-pregnancy experimental analogy [32]. Evidence strength differs across candidate mechanisms and clinical outcomes.

#### Vascular memory after preeclampsia

A history of PE is associated with later chronic hypertension and cardiovascular events [33, 198]. Active vascular reprogramming may contribute beyond shared baseline risk [199]. Maternal leukocytes and vascular tissues show epigenetic and functional changes around affected pregnancies [17, 197, 200]. Human evidence supports broad remodeling more strongly than single-promoter explanations. Persistent fibrosis, smooth-muscle hypertrophy, impaired repair, and altered vascular compliance are plausible contributors [30, 31]. Within PMAFC, these changes may represent incomplete return to the pre-pregnancy reserve state.

#### Metabolic memory after gestational diabetes mellitus

GDM is associated with a substantially increased later risk of type 2 diabetes, although absolute risk varies across populations and follow-up periods [34, 35, 201]. This trajectory may combine pre-existing metabolic vulnerability with pregnancy-imposed demand. Persistent β-cell stress and incomplete recovery of insulin secretory capacity are biologically plausible [42]. Dedifferentiation-like programs remain established mainly in experimental diabetes models [127]. Low-grade inflammation has been described after GDM [202]. Trained-immunity-like reprogramming remains an emerging hypothesis supported mainly by broader reviews and experimental adipose-tissue models [29, 32].

PMAFC integrates these observations without assigning one universal postpartum mechanism. Some women may retain vascular, metabolic, or inflammatory changes after near-threshold or failed adaptation [9, 28]. Prospective studies must separate pre-existing vulnerability from changes caused or amplified by pregnancy. This distinction is essential before pregnancy memory can inform preventive interventions.

## Clinical management and translational strategies

Clinical management should remain anchored in established, disease-specific care. PE management integrates symptoms, blood pressure, laboratory findings, fetal status, and gestational age [173]. Delivery should follow established maternal or fetal indications. GDM care includes medical nutrition therapy, glucose monitoring, pharmacological treatment when needed, and appropriate maternal–fetal assessment [176]. HG management includes rehydration, electrolyte correction, thiamine, antiemetics, nutritional support, and prevention of complications [177]. ICP care combines symptom control, biochemical monitoring, obstetric assessment, and guideline-informed delivery planning [161, 162]. Current evidence-based management of these representative PRDs is summarized in Table 2.

**Table 2:** Current evidence-based management of representative PRDs

Disorder	Current evidence-based management	Primary clinical objectives and qualifications	Evidence basis
Preeclampsia (PE)	• Maternal symptom review, blood-pressure trajectory, laboratory assessment, and fetal surveillance • Urgent treatment of acute severe hypertension • Magnesium sulfate for seizure prophylaxis or treatment when indicated • Antenatal corticosteroids when preterm delivery is anticipated and clinically appropriate • Delivery according to gestational age and established maternal or fetal indications • Postpartum blood-pressure assessment within 72 h after delivery for severe hypertension and no later than 7–10 days postpartum for patients without severe hypertension	Prevent severe maternal complications while balancing fetal maturity. Delivery remains the definitive treatment; biomarker results should not independently determine delivery timing	**Guideline-supported/current care **[173, 203–205]
Gestational diabetes mellitus (GDM)	• Medical nutrition therapy and physical activity when appropriate • Self-monitoring of blood glucose and individualized glycemic targets • Pharmacological treatment when targets are not achieved, with agent selection according to guideline and local practice • Maternal–fetal assessment according to glycemic control and comorbidity • A 75-g OGTT at 4–12 weeks postpartum, followed by continued glycemic surveillance according to test results and individual risk	Achieve glycemic control and reduce maternal and neonatal complications. The 24–28-week OGTT is a diagnostic test and should not be interpreted as a direct measure of maternal metabolic reserve	**Guideline-supported/current care **[176, 206]
Hyperemesis gravidarum (HG)	• Oral or intravenous fluid and electrolyte replacement • Thiamine supplementation, particularly before carbohydrate or dextrose administration when deficiency is possible • Stepwise antiemetic treatment • Nutritional support when oral intake remains inadequate • Assessment and prevention of complications, including thromboprophylaxis when clinically indicated	Restore hydration and nutrition, control symptoms, and prevent maternal complications. GDF15-GFRAL-directed therapy remains investigational and is not part of current care	**Guideline-supported/current care **[177, 207]
Intrahepatic cholestasis of pregnancy (ICP)	• Assessment of pruritus, total bile acids, and liver biochemical tests, with evaluation for alternative liver disease • Ursodeoxycholic acid for maternal pruritus and biochemical improvement • Maternal–fetal assessment according to locally adopted protocols • Delivery planning based on peak bile-acid concentration, gestational age, comorbidity, and guideline recommendations • Repeat liver function tests and total bile acid concentrations at the 6-week postpartum visit to confirm biochemical resolution; persistent pruritus or biochemical abnormalities should prompt further hepatobiliary evaluation	Relieve maternal symptoms and reduce fetal risk through risk-informed obstetric management. Total bile acids are disease-activity markers and not measures of maternal hepatic reserve	**Guideline-supported/current care **[161, 162]

Management and delivery decisions remain based on integrated maternal and fetal assessment and locally adopted guidelines. A formal electronic health record handover should document the pregnancy complication, unresolved abnormalities, current medications, scheduled investigations, and the clinician responsible for follow-up. Maternal–fetal medicine or obstetric services should transfer longer-term care to primary care, with risk-based referral to cardiology, endocrinology, or hepatology services. Cardio-obstetric pathways and dedicated women's cardiovascular clinics are reserved for selected high-risk patients

*GDM* gestational diabetes mellitus, *HG* hyperemesis gravidarum, *ICP* intrahepatic cholestasis of pregnancy, *OGTT* oral glucose tolerance test, *PE* preeclampsia

Emerging biomarkers and mechanism-directed therapies should complement rather than replace established care. Translation requires pregnancy-specific safety assessment, analytical and clinical validation, and incremental utility beyond existing models. It also requires improvement in outcomes important to pregnant women and their infants. Mechanistic plausibility or biomarker modification alone does not establish clinical benefit. We therefore separate established care, selected clinical adjuncts, investigational biomarkers, limited pilot interventions, and preclinical concepts (Fig. 6).

**Fig. 6:**
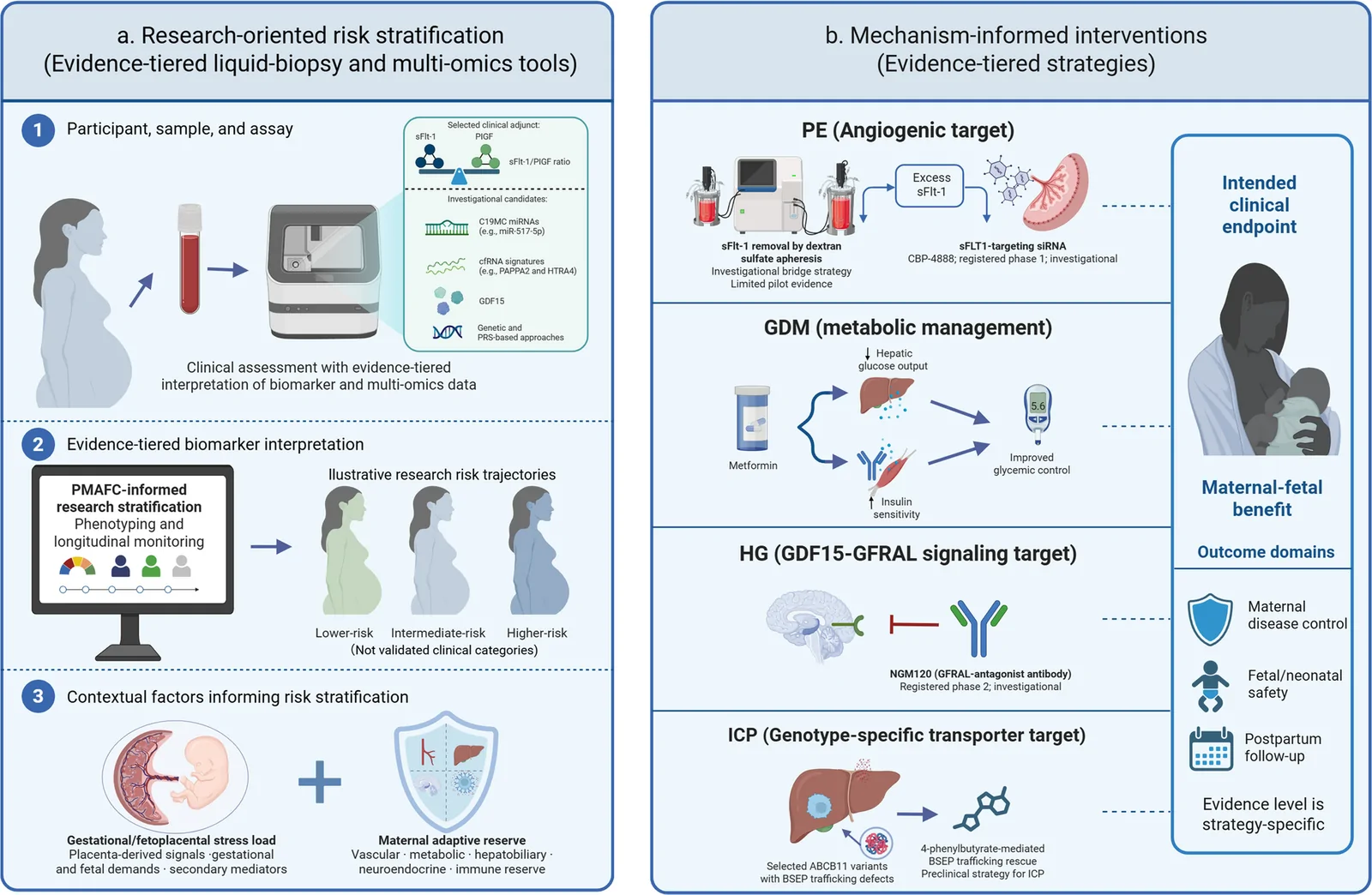
Evidence tiers for risk stratification and mechanism-informed interventions. **a** The sFlt-1/PlGF ratio is a clinical adjunct in selected PE pathways. cfRNA signatures, C19MC miRNAs, GDF15, genetic/PRS approaches and multi-omics models remain investigational. No biomarker or model shown should be used in isolation to determine diagnosis, admission, treatment or timing of delivery. PMAFC-informed research stratification considers gestational/fetoplacental stress load together with maternal adaptive reserve. The illustrated risk trajectories do not represent validated clinical categories. **b** Dextran sulfate apheresis has limited human pilot evidence as an investigational bridge strategy in PE. CBP-4888 is an sFLT1-targeting siRNA undergoing phase 1 clinical evaluation in preterm PE. NGM120 is a GFRAL antagonist antibody undergoing phase 2 clinical evaluation in HG. Metformin is used clinically in selected GDM settings, whereas 4-phenylbutyrate-mediated BSEP trafficking rescue remains a preclinical strategy for ICP. The maternal–fetal endpoint represents an intended outcome rather than a demonstrated common benefit; evidence levels are strategy-specific. Abbreviations: BSEP, bile salt export pump; C19MC, chromosome 19 microRNA cluster; cfRNA, cell-free RNA; GDF15, growth differentiation factor 15; GDM, gestational diabetes mellitus; GFRAL, GDNF family receptor alpha-like; HG, hyperemesis gravidarum; ICP, intrahepatic cholestasis of pregnancy; PE, preeclampsia; PlGF, placental growth factor; PMAFC, Placental-Maternal Adaptation Failure Continuum; PRS, polygenic risk score; sFlt-1, soluble fms-like tyrosine kinase 1; siRNA, small interfering RNA. The figures were created using BioRender.com

### Early prediction and mechanism-linked biomarkers

Established tests and emerging molecular assays have different clinical roles. The oral glucose tolerance test remains central to GDM diagnosis [176, 208]. Uterine artery Doppler and angiogenic biomarkers may support PE assessment in selected populations and pathways [209–211]. Circulating cell-free RNA (cfRNA), placenta-enriched microRNAs, and polygenic risk scores (PRSs) are being studied for earlier risk stratification [212–215]. Their use is constrained by incomplete external validation, assay variability, population transferability, and uncertain incremental utility.

An evidence-tiered interpretation is essential. The sFlt-1/PlGF ratio is a clinical adjunct in selected PE pathways [174, 209, 210]. cfRNA signatures and chromosome 19 microRNA cluster (C19MC) microRNAs remain investigational [212–214, 216]. PRSs are ancestry-dependent research tools [215, 217]. Molecular results should be integrated with established clinical assessment. They should not independently determine treatment, admission, surveillance intensity, or delivery [173, 174, 210].

#### The sFlt-1/PlGF Ratio: clinical interpretation and exploratory phenotyping

The sFlt-1/PlGF ratio is among the most extensively evaluated angiogenic biomarkers in suspected PE. A low ratio has strong short-term rule-out value in appropriately selected populations [209]. Higher ratios may support diagnostic assessment and short-term risk stratification [174, 210, 218]. However, thresholds and predictive performance vary with gestational age, assay platform, population, and clinical indication.

Both components change physiologically across gestation, making interpretation gestation- and assay-dependent [174, 210, 218]. The ratio should be integrated with symptoms, blood pressure, laboratory findings, fetal assessment, and locally validated protocols [173, 174, 210]. It should not independently determine delivery timing.

Combined angiogenic, uterine artery Doppler, and maternal hemodynamic assessment may support exploratory mechanistic phenotyping [175, 211]. A markedly abnormal angiogenic profile may indicate a larger placenta-associated contribution. A modest angiogenic disturbance with impaired maternal cardiovascular adaptation may indicate a larger maternal contribution [175]. These patterns are proposed phenotypes, not validated clinical endotypes.

These data may inform multidisciplinary risk assessment, surveillance, and referral. They should not replace established maternal or fetal indications for escalation or delivery [173, 174, 210]. Within PMAFC, the ratio is a complementary mechanism-linked marker rather than a universal stand-alone threshold.

#### Circulating cfRNA and C19MC microRNAs

Maternal plasma cfRNA contains pregnancy-associated and placenta-enriched transcripts. PE-associated transcriptomic signatures have been detected before clinical onset [212, 213, 219]. Placenta-enriched transcripts, including PAPPA2 and HTRA4, have also been studied as candidate markers [213, 220, 221]. Individual transcript or protein findings are not equivalent to externally validated composite signatures.

C19MC microRNAs are another investigational class of placenta-enriched biomarkers. First-trimester studies have evaluated circulating C19MC microRNAs, including miR-517-5p, for predicting PE and fetal growth restriction [216]. Neither individual C19MC microRNAs nor composite cfRNA signatures are established diagnostic tools.

Translation is limited by pre-analytical variability, RNA instability, processing effects, maternal blood-cell contamination, low signal abundance, and gestational confounding [212–214]. Bioinformatic deconvolution and cross-platform reproducibility are additional challenges. Prospective multicenter studies must demonstrate analytical validity, external performance, and added clinical utility. These assays currently belong in research stratification, biological phenotyping, and trial enrichment.

#### Polygenic risk scores

PRSs combine effects from multiple common variants and have been evaluated mainly as adjuncts for GDM risk prediction. Studies in Chinese and South Asian populations suggest modest gains beyond maternal age and body mass index [217, 222, 223]. Effect estimates, calibration, and transferability vary across ancestry groups [215].

PRSs remain investigational risk-enrichment tools. Their stand-alone predictive value is generally insufficient for treatment decisions. They should not independently trigger nutritional, pharmacological, or obstetric interventions.

HG-associated variants include loci near GDF15 and IGFBP7 and variants in RYR2 [22, 143, 177, 178]. These associations support antecedent susceptibility but do not constitute a validated HG-specific PRS. They also do not justify antiemetic treatment before symptoms develop. Prospective studies must demonstrate added value beyond clinical risk factors.

### Mechanism-based therapeutics: evidence tiers and pregnancy-specific constraints

Mechanism-based strategies span established treatments with additional biological hypotheses, limited pilot interventions, registered clinical studies, and preclinical molecular approaches. Each strategy should be interpreted according to its evidence tier. Representative mechanism-based strategies and their evidence tiers are summarized in Table 3.

**Table 3:** Emerging and investigational mechanism-based interventions within the PMAFC framework

Disorder/target population	Intervention	Proposed target or mechanism	Evidence tier	Current evidence and principal limitation	Key references
Severe preterm PE with marked angiogenic imbalance	**Dextran sulfate apheresis**	Extracorporeal removal of circulating sFlt-1	**2. Human proof-of-concept**	Small, nonrandomized clinical studies reported sFlt-1 reduction, lower proteinuria, and pregnancy prolongation. Apheresis remains an investigational bridge strategy	[224, 225]
sFLT1-mediated preterm PE	**CBP-4888**	siRNA-mediated reduction of placental sFLT1 expression	**3. Registered clinical trial**	Phase 1 open-label dose-finding study in hospitalized participants with preterm PE receiving standard-of-care expectant management. The study assesses safety, tolerability, pharmacokinetics, pharmacodynamic effects on maternal plasma sFLT1, and maternal and infant outcomes	[226, 227]
Experimental PE models	**Placenta-directed sFLT1 RNAi platforms**	Silencing of placental sFLT1 production	**4. Preclinical/experimental**	Reduced circulating sFLT1in pregnant mice and attenuated PE-like features in a baboon model. Human studies should assess efficacy, fetal exposure, and developmental safety	[228]
Severe nausea and vomiting/HG	**NGM120**	GFRAL-antagonist monoclonal antibody that blocks GDF15 signaling	**3. Registered clinical trial**	Phase 2 randomized, placebo-controlled study in pregnant participants with severe nausea and vomiting (HG). The study assesses safety and tolerability in addition to standard and supportive care, together with changes in PUQE-24 and HELP scores	[229]
GDF15-related emetic pathway in HG	**Ponsegromab**	Neutralization of circulating GDF15	**5. Evidence from another disease context only**	Human phase 2 evidence is from cancer cachexia, not HG or pregnancy. Direct extrapolation to maternal–fetal safety or symptom control in HG is not justified	[230]
Selected ABCB11/BSEP trafficking-defective cholestatic disease	**4-Phenylbutyrate and related chaperone strategies**	Improved BSEP processing, trafficking, canalicular expression, and transport	**5. Evidence from another disease context only**	Evidence derives from in vitro models and selected PFIC2 cases. Application to ICP remains an experimental strategy	[156, 231]
GDM/proposed PE-modifying use	**Metformin beyond glycemic control**	Insulin sensitization and proposed reduction of sFlt-1 and soluble endoglin secretion	**4. Preclinical/experimental**	Metformin is used for glycemic management in selected GDM settings, its proposed disease-modifying use in PE remains investigational	[232, 233]
GDM prevention in at-risk pregnancy	**Myo-inositol**	Modulation of insulin second-messenger signaling	**2. Human proof-of-concept**	Randomized trials and meta-analyses suggest a possible reduction in GDM incidence, but heterogeneity in formulation, dose, population, and diagnostic criteria limits generalizability	[234–236]

Evidence tier applies to the specific mechanism-based use shown: 1, guideline-supported/current care; 2, human proof-of-concept; 3, registered clinical trial; 4, preclinical/experimental; and 5, evidence from another disease context only. No intervention in Table 3 is guideline-supported or part of current care for the mechanism-specific use presented. The tier assigned to an intervention may differ from its status in another indication; for example, metformin is established for glycemic management in selected GDM settings but remains experimental as a PE disease-modifying strategy

*ABCB11* ATP-binding cassette subfamily B member 11, *BSEP* bile salt export pump, *GDF15* growth differentiation factor 15, *GFRAL* GDNF family receptor alpha-like, *GDM* gestational diabetes mellitus, *HG* hyperemesis gravidarum, *HELP* HyperEmesis Level Prediction, *ICP* intrahepatic cholestasis of pregnancy, *PE* preeclampsia, *PFIC2* progressive familial intrahepatic cholestasis type 2, *PMAFC* Placental-Maternal Adaptation Failure Continuum, *siRNA* small interfering RNA, *sFlt-1/sFLT1* soluble fms-like tyrosine kinase-1

Pregnancy alters plasma volume, albumin, protein binding, renal perfusion, glomerular filtration, hepatic enzymes, and transporter expression [237, 238]. These changes can alter drug exposure across gestation. Evaluation of siRNA and antibody therapies must address placental transfer, maternal–fetal exposure, tissue distribution, immunogenicity, developmental toxicity, and lactational exposure [228, 237–239].

Translation requires a defined evidence tier, mechanistically justified selection, pregnancy-specific pharmacology, and clinically meaningful benefit [237–239]. For strategies intended to prolong pregnancy, potential benefit must be balanced against maternal and fetal safety. Such strategies must never override established indications for delivery [173, 224, 225] (Fig. 6b).

#### Reducing placenta-associated sFlt-1 burden in preeclampsia

In severe preterm PE with marked angiogenic imbalance, sFlt-1 removal has been studied as a possible bridge strategy. A small open-label pilot study used dextran sulfate apheresis and reported lower sFlt-1, reduced proteinuria, and pregnancy prolongation [224]. The absence of randomization and the intensive selection of participants limit causal interpretation and generalizability [224, 225]. Apheresis remains an investigational bridge strategy.

Placenta-directed RNA interference targets placental sFLT1 production. Modified siRNAs reduced circulating sFLT1 in pregnant mice and attenuated PE-like features in a baboon model [228]. CBP-4888 is a sFLT1-targeting siRNA undergoing a phase 1 open-label, dose-finding study in hospitalized participants with preterm PE receiving standard-of-care expectant management. The study is designed to assess safety, tolerability, pharmacokinetics, pharmacodynamic effects on maternal plasma sFLT1, and maternal and infant outcomes [226, 227].

Future studies must define gestational windows, molecular selection criteria, dose-exposure relationships, fetal exposure, immunogenicity, and meaningful outcomes. Neither apheresis nor sFLT1-directed RNA interference should replace established management or indications for delivery [173].

#### Metabolic management and investigational strategies in gestational diabetes mellitus

GDM management remains centered on medical nutrition therapy, appropriate physical activity, glucose monitoring, and pharmacological treatment when targets are unmet [176]. Mechanistic findings should be interpreted within this established clinical pathway.

Metformin may have biological effects beyond glucose lowering. Experimental studies report reduced trophoblast and endothelial secretion of sFlt-1 and soluble endoglin [232, 233]. Clinical studies have compared metformin with insulin for GDM outcomes [240, 241]. The proposed disease-modifying use of metformin in PE remains investigational. Heterogeneity in populations, treatment selection, and background care also limits comparisons.

Meta-analyses of randomized trials associate myo-inositol supplementation with lower GDM incidence [234–236]. Heterogeneity in dose, formulation, selection, diagnostic criteria, and background care limits generalizability. Myo-inositol is therefore a potential preventive adjunct rather than an established mechanism-based intervention.

Future trials should assess glycemia, gestational weight, hypertensive disorders, fetal growth, neonatal outcomes, and longer-term maternal metabolism. Molecular or intermediate metabolic endpoints alone are insufficient.

#### GDF15-GFRAL-directed approaches in hyperemesis gravidarum

HG care remains based on rehydration, electrolyte correction, thiamine, antiemetics, nutritional support, and complication prevention [177]. The GDF15-GFRAL pathway is a potential target for severe or refractory disease. It does not replace established supportive and pharmacological management [22, 144, 145].

Fetoplacental GDF15 signals through the GFRAL-RET complex in the hindbrain [22, 144, 145]. This pathway supports a role in pregnancy-associated nausea, vomiting, and altered intake. Ponsegromab neutralized GDF15 and showed clinical effects in nonpregnant patients with cancer cachexia [230]. Those findings cannot be extrapolated directly to HG because the population, disease context, and pregnancy risks differ.

NGM120, a GFRAL-antagonist monoclonal antibody, is being evaluated in the phase 2 randomized, placebo-controlled EMERALD study in pregnant participants with severe nausea and vomiting (HG). The study assesses safety and tolerability in addition to standard and supportive care, together with changes in validated symptom measures including PUQE-24 and HELP scores [229].

#### Transporter-rescue concepts in intrahepatic cholestasis of pregnancy

ICP is genetically and mechanistically heterogeneous, and management should follow established recommendations [161, 162]. Variants in hepatobiliary transporter genes, including ABCB11, contribute to susceptibility in selected patients but do not explain all cases [170].

Chemical chaperones have been studied for selected trafficking-defective ABCB11 variants. In experimental models and inherited BSEP deficiency, 4-phenylbutyrate can improve processing, trafficking, or canalicular expression of selected variants [156, 231]. Application of 4-phenylbutyrate to ICP therefore remains experimental.

Transporter rescue remains a genotype-dependent preclinical concept. Translation would require molecular confirmation, pregnancy-specific pharmacology, maternal–fetal safety assessment, and benefit beyond biochemical correction.

### Postpartum management and the fourth trimester

Risks associated with PRDs may extend beyond delivery. PE is associated with later cardiovascular disease [203, 242, 243], whereas GDM is associated with subsequent type 2 diabetes and broader cardiometabolic disease [34, 35, 201]. Absolute risks vary according to baseline susceptibility, disease severity and recurrence, postpartum exposures, and follow-up. PMAFC may help organize these observations but is not a validated postpartum risk-prediction model.

After PE or gestational hypertension, blood pressure should be evaluated within 72 h after delivery in patients with severe hypertension and no later than 7–10 days postpartum in those without severe hypertension [203, 204, 242]. Medication review and transition to longer-term cardiovascular risk assessment are also important. Cardio-obstetric pathways and dedicated women's cardiovascular clinics may support selected high-risk patients [244].

After GDM, a 75-g oral glucose tolerance test should be performed at 4–12 weeks postpartum [176, 206]. Further glycemic surveillance should be determined by the test results and individual risk. Blood pressure, body weight, lipid profile, physical activity, and other cardiometabolic factors should also be assessed as appropriate [203, 206].

After HG, follow-up should confirm correction of dehydration, nutritional deficiencies, electrolyte disturbances, and persistent psychological or functional effects [177]. After ICP, liver function tests and serum total bile acid concentrations should be repeated at the 6-week postpartum visit to confirm biochemical resolution [161, 162]. Persistent pruritus or abnormal liver biochemistry or bile acid concentrations at that time should prompt further hepatobiliary evaluation.

Continuity of care should be supported by a structured transition process. Before discharge or during the early postpartum review, a formal electronic health record handover should document the pregnancy complication, its severity and recurrence, unresolved abnormalities, current medications, scheduled investigations, and the clinician responsible for follow-up. Maternal–fetal medicine or obstetric services should transfer longer-term care to primary care and, according to the disorder, test results, and individual risk, arrange referral to cardiovascular, endocrinology, or hepatology services. Cardio-obstetric pathways, Pregnancy Heart Teams, and dedicated women’s cardiovascular clinics may support selected high-risk patients but should not be considered universal requirements [203, 205, 244]. Following the initial postpartum assessment, additional risk-stratified reviews at 6 and 12 months may include blood pressure, body weight, glycemic status, lipid profile, and clinically indicated liver biochemistry, with subsequent follow-up intervals determined by findings and baseline risk [203, 205, 206, 244].

Longer or more intensive lactation is observationally associated with a lower risk of type 2 diabetes after GDM [245–247]. This association does not establish causality, because lactation initiation and duration are influenced by social, metabolic, obstetric, and behavioural factors.

Pharmacological modification of long-term postpartum risk remains investigational. A postnatal enalapril pilot study and experimental statin studies provide hypothesis-generating evidence [248, 249], but do not establish routine disease-modifying treatment. Postpartum care should instead prioritize completion of scheduled surveillance, management of modifiable risk factors, continuity of care, and preparation for future pregnancies [203, 205, 244].

### Implementation in low- and middle-income settings

Implementation priorities include clinical benefit, affordability, feasibility, and linkage to effective care. Molecular assays should complement rather than displace antenatal assessment, blood-pressure measurement, glucose testing, symptom evaluation, and referral pathways.

An evidence-tiered pathway begins with broadly applicable assessment and disease-specific screening. A second tier may use locally validated angiogenic testing in selected PE pathways when results can guide referral or surveillance [174, 210]. cfRNA and C19MC assays should remain in research settings [212–214, 216]. PRSs and multi-omics models require population-specific calibration, external validation, and transferability assessment [215, 217].

Implementation studies should examine sample stability, turnaround, equipment, training, cold-chain dependence, population characteristics, referral capacity, and confirmatory assessment [214, 215]. A molecular result has little clinical value without timely review, surveillance, referral, and treatment.

PMAFC-informed pathways should be scalable and evidence-tiered. Equitable evaluation requires diverse populations and assessment of access, clinical outcomes, and health-system efficiency.

Translational evidence remains heterogeneous. The sFlt-1/PlGF ratio is a selected clinical adjunct [174, 209, 210]. cfRNA and C19MC microRNAs remain investigational [212–214, 216], and PRSs have limited stand-alone utility [215, 217]. Apheresis has limited pilot evidence [224, 225], sFLT1 silencing is preclinical or in registered early-phase evaluation [226–228]. GFRAL antagonism is under clinical investigation [229], and transporter rescue remains preclinical [156, 231]. Studies should prioritize safety, patient-important outcomes, external validation, economics, and equity.

## Conclusion

Pregnancy is not a single physiological stress but a coordinated test of vascular, metabolic, immune, neuroendocrine, and hepatobiliary adaptation. PE, GDM, HG, and ICP remain clinically distinct disorders with different proximal mechanisms and management requirements. The PMAFC framework does not propose a shared disease origin. Instead, it offers a common adaptive logic in which gestational and fetoplacental stress interacts with the reserve available across maternal organ systems. This distinction enables mechanistic comparison while preserving diagnostic boundaries and disease-specific causal architecture.

Within this framework, cross-disorder associations are interpreted as changes in adaptive context rather than as fixed disease sequences. An established disorder may generate secondary vascular, metabolic, hepatic, inflammatory, or neuroendocrine stress that narrows the reserve margin in another domain. Pre-existing vulnerability may likewise amplify the effects of pregnancy-associated signals before a clinical phenotype becomes apparent. Such interactions may produce sensitization, amplification, or bidirectional crosstalk without making the disorders interchangeable. This relational view also separates antecedent vulnerability from downstream biomarkers and consequences of established disease.

PMAFC should nevertheless be regarded as a heuristic framework rather than a validated diagnostic, prognostic, or therapeutic model. The proposed load and reserve domains are not yet represented by standardized clinical measures, and their relative contributions may vary across gestation and populations. Current biomarkers may reflect exposure, reserve, disease activity, or downstream injury, and these roles cannot be inferred from cross-sectional associations alone. Apparent overlap between disorders may also arise from shared risk factors, treatment effects, or ascertainment. Temporal ordering is difficult to establish because susceptibility, disease severity, treatment, and secondary physiological changes can influence the same pathways. The framework should therefore complement, not replace, disease-specific models, diagnostic criteria, or guideline-based management. Evidence should remain tiered so that established clinical tools are clearly separated from exploratory biomarkers and mechanism-informed interventions. The value of PMAFC will depend on whether it generates falsifiable hypotheses and adds explanatory or predictive information beyond simpler approaches.

Testing PMAFC will require longitudinal studies beginning before disease onset, with repeated assessment of candidate stress load and reserve measures across pregnancy and postpartum. Interaction models should be compared with additive models and established disease-specific predictors, then externally validated across populations with diverse ancestral backgrounds and maternal comorbidity profiles, as well as across healthcare settings. Studies should prespecify maternal and fetal outcomes, assess model calibration and clinical utility, and determine whether any improvement persists after adjustment for conventional risk factors. Validation should determine which measures genuinely distinguish adaptive reserve from disease activity and whether dynamic reserve trajectories identify reversible stages of maladaptation. It should also test whether interventions can reduce stress load or preserve reserve without compromising maternal or fetal safety. Postpartum studies should determine whether different antenatal pathways leave distinct or convergent maternal imprints. Until these questions are resolved, clinical care should remain disease-specific and guideline-based. The immediate role of PMAFC is to organize mechanisms, sharpen cross-disorder questions, and define where shared biology can be investigated without being mistaken for shared causality.

## Data Availability

Data sharing is not applicable to this review because no datasets were generated or analyzed.
